# Residential childcare worker perceptions of work-related achievement and pride

**DOI:** 10.3389/frcha.2024.1360365

**Published:** 2024-07-25

**Authors:** Denise Michelle Brend, Oyeniyi Samuel Olaniyan, Delphine Collin-Vézina

**Affiliations:** ^1^ School of Social Work and Criminology, Laval University, Quebec City, QC, Canada; ^2^ Department of Psychology, Inland Norway University of Applied Sciences, Lillehammer, Norway; ^3^ School of Social Work, McGill University, Montreal, QC, Canada

**Keywords:** childcare worker, wellbeing, pride, accomplishment, child protection, youth justice, residential care, protective factor

## Abstract

**Introduction:**

Multiple risk and protective factors influence the wellbeing and retention of child protective and youth justice professionals. Less attention has been given to empirically understand how residential childcare workers (RCW) experience these factors. A sense of pride and of achievement may be related to competence and satisfaction, which have been identified as protective factors against staff turnover.

**Methods:**

Responses to the Secure Base Interview Protocol question “What aspects of caring for (name of child in their care) have given you the greatest sense of pride or achievement?” were extracted from individual interview transcripts from Canadian RCW and analyzed using the Interpretive Description methodology. Themes were aggregated using the thematic analysis technique to create descriptions of RCW pride and achievement.

**Results:**

The RCW identified many experiences of work-related pride and achievement while caring for children and youth. These positive experiences were described to occur contingent on the level of mutuality and trust in the helping relationship shared between the RCW and child or youth in their care.

**Discussion:**

The reciprocal nature of the relationships described by RCW that gave rise to their felt sense of pride and accomplishment is a novel finding. Future work is indicated to better understand how protective factors related to RCW wellbeing may indeed be relationally constructed and dependent.

## Introduction

There is a pressing need to identify experiences that promote professional wellbeing and resilience among residential childcare workers (RCW). Research examining caring for children and youth in placement under child protective or youth justice mandates portrays a profession disproportionately impacted by burnout, stress, and turnover. Recent findings show that the impact of this work is similar across different geographical locations and cultural practices (1–4). Residential childcare work is characterized by constant pressure, high workload, and an important emotional burden; and, workplace risks like burnout, secondary traumatic stress (i.e., vicarious trauma, compassion fatigue), and moral distress, are in most cases connected to different aspects of the work environment far beyond the control of individual workers. The World Health Organization (WHO), for example, described burnout as symptoms stemming from workplace stressors that have not been properly managed (5). Research has also shown that professionals can exhibit similar symptoms to their clients who have experienced firsthand trauma (6, 7). Importantly, the diminished wellbeing of professionals has been linked to poorer outcomes for the children and youth in care (8–10). The wellbeing of RCW is, therefore, a critical aspect of effective service delivery.

The past decade has witnessed a surge in research identifying risk and protective factors related to the impacts of working in child welfare (11–18). Early study framed resilience as an individual strength, capacity, or character trait, i.e., an ability to withstand hardship. Recently, it has been defined by Yoon et al. (19) as the product of a complex interplay between multiple factors over time, with a structure and development that can be variable. Their resilience theoretical framework reflects this complexity by adopting “a strengths-based lens to understand the protective factors, processes, and pathways through which individuals achieve adaptive functioning in the face of adversity” [(19), p. 2]. This is consistent with the theorizing of a growing body of resilience researchers, including those looking at organizational resiliency models adopted in and across professional settings to engage the environment in the promotion of wellbeing for staff (20–22).

Most of the exposures to workplace risk are embedded within the tasks that define RCW work, it is not possible to separate these risks from the everyday work. In the face of unavoidable exposures to workplace risk, routine access to, and the promotion of protective factors is critical towards RCW wellbeing. The systematic review of vicarious traumatization among child protection workers by Molnar et al. (23) highlights risk factors including personal trauma, work-family conflict, exposure to vicarious trauma, job overload, exposure to disturbing media, role-related factors, and lack of resources; protective factors included supervisor support, co-worker support, positive social support outside of work, age, and job satisfaction. In their recent study on increasing retention and the promotion of resilience in the field of child protection, Russ et al. (16) interviewed 24 Australian child protection workers over two data collection phases within a 12-month period. They found that participants described their work as very challenging, difficult, and complicated. They also reported high stress, direct trauma, and vicarious trauma. In the face of these many challenges attached to their roles, most of the participants also reported marked job commitment, self-awareness, self-efficacy and agency, use of reflective practices, and the application of relationship-based approaches to work. Authors concluded that these findings point to the importance of resilience among this work group (16). In their study of evidence-informed resiliency, Kalergis and Anderson (21) interviewed ten child abuse leaders finding that the conscious practice of an organizational resilience model (ORM) led to the promotion of a healthy and resilient workforce. They emphasized the effectiveness of the ORM if carefully adopted and implemented in child welfare organizations. The elements of the ORM are self-knowledge and insight; sense of hope; healthy coping; strong relationships; personal perspective and meaning. Each of these elements is to be applied at the personal, professional, and organizational levels (21). Therefore, a sense of pride in one's work and personal accomplishment might be protective factors linked to both self-knowledge and insight (knowing what I find gratifying) and personal perspective and meaning (what it means to me to be accomplished at work).

Previous research suggests that a strong feeling of personal accomplishment and pride could promote identity and loyalty towards organizations (24, 25). What identity, personal accomplishment, or factors intrinsic to feeling pride are for RCW has yet to be elaborated. Further, how one feels about trauma-exposure has also been shown to influence the severity of post-traumatic stress (26). Painful feelings, such as betrayal, can increase symptom severity. Indeed, betrayal trauma can be perpetrated by individuals, organization or institutions (27). Li et al. (28) investigated organizational climate, job satisfaction, and turnover among 849 voluntary child welfare workers, finding that “diverse individual characteristics together with stressful, unjust, exclusionary and non-supportive organizational climate negatively influence individual wellbeing and [led] to lack of job satisfaction and lower organizational commitment, which in turn [led] to stronger intentions to leave the job” (p.566). It appears that protective factors, including the experience of pride or accomplishment or job satisfaction more broadly, must be considered within the local organizational context.

Despite the array of research that has explored different protective factors in the field, only a handful of research has examined factors relating to a sense of accomplishment or pride when providing day-to-day care for children and youth in placement. For example, Rycraft (29) investigated retention factors among a 23 child welfare workers and found that a “sense of mission” was protective. Datum such as, “I feel I am doing something of value” suggests a potential overlap between mission and pride. Mor Barak et al. (30) tested “an expanded version of a theoretical model depicting the relationship between diversity characteristics, organizational climate and personal outcome variables on intention to leave” among 418 child welfare workers (p. 553). Among the subsample of participants who agreed to participate in an in-depth interview (*n* = 33), they found that the majority, “regardless of personal characteristics, expressed satisfaction and pride in having a positive impact and in making a difference in their clients’ lives” (565). How these factors might mitigate workplace risks to RCW remains to be examined.

The theoretical framework we will be adopting is embedded within the model of organizational social context (OSC) (31). Social contexts can be described as the relationship quality between a network of individuals, characterized by expectations, norms, as well as individual behavior that is nested in shared perceptions (32, 33). The importance of the social context cannot be overemphasized as studies have shown its connection to various other important organizational factors (31, 34–36). Findings have shown that the degree to which workers stay committed and engaged at work, as well as the quality of service they deliver, are connected to the quality of the organizational social context (37–40). In their study of OSC, Glisson et al. (31) identified personal achievement as a dimension of one of the three sub-categories of OSC. The feeling of achievement and accomplishment was related to engagement and described as a feeling of being successful and as establishing a satisfied level of professional attainment. Recent research has also suggested that subjective experience of accomplishment may be related to a sense of competence or self-efficacy (41, 42) and an absence of burnout (43). The subjective experience of pride in one's work may also be related to job satisfaction and moral distress (1, 44). The management of exposure to, and recovery from, potentially traumatic or distressing workplace experiences appears to have an important relationship with individual meaning-making (45, 46). Indeed, damage to the capacity to mentalize, or make-sense of the inner worlds the self and others, is an important impediment to psychological wellbeing (47). To better understand how these factors might be experienced by RCW and to identify factors that may only be visible within their lived experience qualitative investigation is indicated. The objective of this study was to describe RCW experiences of accomplishment or pride in the course of working with individual children or youth placed in rehabilitation centers under youth protective or justice mandates, through an OSC lens.

## Method

### Participants and procedure

The present study occurred within a larger longitudinal partnered research project with residential treatment centers in child welfare and youth justice contexts across the province of Quebec. The project was approved by the [anonymized] research ethics board, and the associated integrated health and social service centers administering each site. Interviews were conducted with a randomized sample of frontline and clinical staff across the participating institutions. Eighty-one RCW accepted to participate in these interviews. The data included in this analysis were extracted from the time-1 interviews taken at baseline, prior to the implementation of a trauma-informed care program. All interviews were conducted in-person at the place of employment of the participant during their paid working hours and in a sound proofed and private room and audio recorded. Two research professionals and one postdoctoral scholar conducted the interviews, each had experience with both youth protection and young offenders service providers, and each was selected by the second author for their goodness of fit in this role. The recordings were transcribed by professional transcribers. The interviews were structured by the Secure Base Interview Protocol (48) and the unit of analysis for this study was RCW responses to the question, “What aspects of caring for (name of child in their care) have given you the greatest sense of pride or achievement?”. As reported in [anonymized], all interviews were conducted and analyzed in French (the maternal language of all participants). Extracts of the data included in English publications were first translated using AI tools (Deepl and ChatGPT) and then refined by the first and third authors whose maternal languages are French and English respectively.

Table 1 provides more details of the participants in the present study.

**Table 1:** Participant demographics and workplace context*s.*

Variable	*n*	% or m	SD	Range
Worker gender				
Female	56	69.1%		
Male	25	30.9%		
Worker age^a^	80	37.4	8.1	23–55
Worker highest degree^a^				
Community College	50	62.5%		
Undergraduate University^b^	30	37.5%		
Worker position^a^				
Frontline staff^a^	76	95.0%		
Clinical support^a^	4	5.0%		
Length of time in position^a^	80	9.2	8.4	0–32
Years of YP/YCJ experience^a^	80	12.4	8.7	0–35
Age of youth in unit				
Pre-adolescent (6–13)^c^	34	43.0%		
Adolescent (11–20)^c^	47	58.0%		
Legal mandate of unit by gender				
Youth criminal justice act–boys	16	19.8%		
Youth protection act (YPA)–boys	22	27.2%		
YPA–girls	17	21.0%		
YPA–girls & boys	26	32.1%		

*n* = 81.

^a^ data missing from one worker.

^b^ two participants held a master’s degree.

^c^ these age categories overlap due to exceptional cases in which siblings were kept together in residential settings despite one (or more) not belonging to the targeted ages served.

The units in which these RCW worked were all rehabilitation centres for children and youth needing intensive services for mental health/behavioral needs, in some cases they also served as permanent placements for youth when no other options were available. Efforts were made in all settings to promote family involvement, school attendance, mental health services and recreational programming. Units generally offer approximately 12 beds, and some suburban and urban units were on campuses with multiple units. Staff worked 8-hour shifts with 1:6 ratios of staff to residents, in rare cases a third staff member was added when a child or youth presented extremely high behavioural management needs. Overnight staff were not included as their role was uniquely to provide surveillance.

### Analytical approach

Data were extracted from individual interview transcripts of 81 Canadian RCW. To explore RCW descriptions and meanings, we employed the interpretive description methodology (49, 50). This methodology was selected as it is an “accessible and theoretically flexible approach to analysing qualitative data …[that] can address complex experiential questions while producing practical outcomes” [ (49), p. 336]. For a more complete description of how this method was conceptualized, see [anonymized]. The 81 interview transcripts were analyzed using Nvivo R14.23.0 (51) following methodological recommendations by Braun and Clarke (52). The first author was familiar with much of the data having conducted almost one third of the interviews and the second author was the principal investigator of the research project within which this analysis was conducted. Both began by familiarizing themselves with the entire data set for this analysis. Then the data was systematically coded by the first author and reviewed by the second author, there was no predetermined code or thematic structure. The 252 resulting codes were then clustered into initial themes which were generated from the coded and collated data. These themes were further refined, re-developed, and reconsidered over multiple reappraisals of the data and eventually names for each that reflected a close read of all of the data were determined. All data was retained and reflected in at least one of the final themes.

Trustworthiness and credibility of findings was increased through the maintenance of training and research relationships with child protective and youth justice services by the first and second authors throughout the analysis of these data. As described in [anonymized], both authors were engaged in ongoing self-reflection, consultation with a network of child welfare and youth justice researchers and practitioners, and neither author had conflicts of interest with the sites or participants in this study. To enrich the interpretation of the data, a third researcher from an organizational psychology background was recruited who had no prior connection to this project. They were invited to re-examine the analysis and consider the findings within a broader organizational context.

## Results

We employed iterative thematic analysis to explore the participants' responses. All of the data was coded, and all of the codes were successfully synthesized into four overarching themes. The smallest of those themes included a subsample of four RCW who were unable to identify a sense of pride or accomplishment in their work with a youth in their primary care. Some of the reasons they expressed for this included a sense that that nothing they tried was working, that their experience of working with this child or youth left them with a feeling of being in a void, and that their role was always to be “part of the bad news.” This brief description encompasses all of the data from the subsample of four RCW who did not report a sense of pride or accomplishment. Three themes synthesize the remaining RCW (*n* = 77) responses. These themes, and the percentage of the data that is represented within each theme are: 1. Relational factors: data speaking to the quality of the connection experienced or of the relationship (56%); 2. Child and youth factors: data describing vicarious pleasure through the child's accomplishments, or the impact of the child's qualities on the RCW (28%); 3. RCW factors, experiences in relation to the abilities or efforts made by individual RCW (16%).

### Theme 1: relational factors

This theme holds the stories of the connections between these RCW and the children and youth in their care. RCW described how the quality of those connections impacted them. Developing or repairing a child's ability to form a secure attachment bond is a fundamental priority in many RCW helping relationships. The possibility of healing attachment bonds rests uniquely within relationships, for example, in the sense of security provided by interactions, shared routines, and rituals with caregivers. In recounting one of her experiences, a participant described how she set up a secure and nurturing environment with one young person,

…at bedtime, I have a special routine with him. I make a drawing on his back with the hairbrush, then I make him a caterpillar cocoonwith his blankets, and I gather him up in his cocoon and rock him…..It is very special. It happened on its own….[one night] he said, “Tuck me in”And that is how I was tucked in when I was little, so I did the same thing,then he asked, “Can you rock me?” It happened just like that and then everynight we did the same thing.

The warmth and care of these interactions was not only pleasurable for the child, but for the RCW as well. Indeed, she went on to explain how this tenderness between them seemed to pave the way for successful interventions.

I have a special bond with this child, in meetings we often try tounderstand why interventions work between the two of us—but not for others…

Other data grouped within this theme includes a worker stating, “I was trusted.” Another RCW reported, “I was a secure base for [this child/youth].” Another simply reported, “I am known.” This RCW described the experience of becoming collaborative with the youth in their care:

How far we've come in a year. Before, it was all about the rules. Now, we have succeeded in working in a different way, which is engaging us…in collaboration. That's it (snaps fingers) that's the word! We can collaborate with her. Reach for her to join us, slowly and gently to help her to become aware of her challenges and to work towards a common goal.

Each of these statements speaks to the reciprocal nature of the helping relationship between these RCW and the child or youth in their care. They describe experiences possible only within an intimate connection with another person who is engaging with them.

### Theme 2: child and youth factors

RCW also felt accomplished through “the growth & accomplishments” of the children and youth in their care. One RCW explained, “Our job is to help them, to help them have successes, and be happy as a family. So, when you feel like you've reached that goal, it's fun”. Others described the pride they felt “seeing them feeling good.” One RCW explained that they felt great pride and accomplishment when they could say that the young person in their care was “loveable.” Given the types of behavioural and emotion regulation challenges demonstrated by children and youth in high care level settings, it is significant for an RCW to be able to feel that the young person in their care was loveable. How RCW can take pleasure in repairing relational functioning is also evident in this account:

We have a special relationship, we eat together and go and get [an ethnic food from another country] all the time, which he introduced me to and which I love now. And he's proud when he sees me, he tells me what he did at school and that he succeeded at such and such a level, and now he's joined his school's sports team.

In these brief lines we can see how this RCW enjoyed learning from this youth, sharing a pleasurable experience together, being exposed to his culture, vicariously enjoying his pride in himself, and witnessing him grow and try new things on his own.

The answer given by this RCW traces the growing capacity of the boy in her care to manage what began as explosive anger,

Um…in relation to [name of boy], what makes me pride is to be able to say… When he arrived here he was a little bomb that exploded…He was the most explosive child I have ever seen, her went from 0 to 10 in 3 s… at the end, when he left us, he was able to go from 0 to 10 in 10 s—but he could get himself back down to 5, and he was able to verbalize “Wow, yeah, that really made me angry!” You know?! He could then say, “I am going to go for a little walk…” He made some incredible progress. I mean, his impulsivity was still there, and his… No, not impulsivity, because he could… he was way better, it was kind of like his first reaction was angry, that wave of anger was still there, but he could manage it differently…You know? He put effort into everything we tried to offer him, in all of it… We really came a long way.

The story of this boy's growth began with a description of his behaviour, then a testimony to his newly developed skills. The RCW reframing his angry reaction from “impulsivity” to a wave of anger he could manage suggests that she no longer saw his anger as a personality trait. Perhaps he had transformed from an impulsive child to a child who had developed the necessary skills to manage his personal challenges. Perhaps she had changed from an RCW who labelled children's symptoms into one who acknowledged their efforts? The progression that this datum indicates in unclear. What is clear is that the quote ended with an acknowledgement that it was not he alone who had progressed—but both of them together.

### Theme 3: RCW factors

The final category of data reflecting RCW pride and accomplishment shows how RCW saw growth in themselves, they used phrases such as: “When I can…” do something challenging; or that “I did not give up”; sometimes they used language of performance, such as “I am improving”; or described strategies that they found to be less emotionally impacted, “I am not too attached.” This RCW met the challenge to remain emotionally attuned with a youth while they were presenting challenging behaviours.

I was able to control myself. The way he speaks, it can really get under your skin, you want to just [indicating what appeared to be frustration]…But I was able to put those feelings aside and work with him, for him.

In the following datum the RCW described being able to stop a youth from falling into a pattern of escalating affect that usually led to violence. Her description shows the complexity of this work. She explains that she understands that this youth has a tendency to repeat this cycle, how it progresses, and how she has identified strategies to interrupt, or even, prevent this from happening. It bears acknowledgement that such processes of assessment and intervention are being done in a context with 6–12 other youth who are simultaneously expressing their own relational needs. Yet, this RCW was able to co-regulate with this individual youth at a time that they were demonstrating high-risk behaviour.

It's when I manage to… To stop his cycle- the escalation of violence… at the very beginning. The final result was that he broke things, he broke his music player and everything. But there are times I'm able to defuse it beforehand, by [distracting him], by taking him out of his, his black bubble…When I'm able to-, to get him out and get him to decompress before, before he starts to think on his own, before he starts to, to see…only darkness.

As seen across the other two themes, the experience of pride or accomplishment was not simply present when things were going smoothly or easily. Indeed, RCW were proud when they could manage the complexity of the situations they were in, the wellbeing of the youth in their care, and their own behaviour and affect.

## Discussion

The present study sought to investigate what pride and accomplishment was for RCW in child protective and youth justice contexts. Perhaps the most interesting finding was the value and quality of the individual helping relationships towards promoting pride and accomplishment. Despite the evident power of significant interpersonal relationships across the lifespan to promote wellbeing, the experience of wellbeing for professionals in helping relationships is less evident. Trauma sustained during early developmental can result in many impacts, including impaired interpersonal empathy and reactive verbal or physical aggression (53). Children and youth with these types of post-traumatic adaptations were evident in many of the accounts in this data set. Unfortunate results of such post-traumatic adaptations include diminished capacities to trust in others, to know how to and want to engage with them. Feeling connected with these youth, collaborating with them, feeling their growth, experiencing one's own growth as a more secure attachment figure—these experiences gave rise to pride and accomplishment. Much has been written about how securely attached caregivers can create the opportunity for insecurely attached clients to heal. Less has considered the nature of secure attachment as potentially bi- or multi-directional. Helping relationships provide the therapeutic space for healing through many mechanisms (mentalization, physical and affective co-regulation, the opportunity to practice interpersonal communication and boundary setting, etc.), perhaps experiencing *being* a secure attachment figure also promotes wellbeing. Indeed, it is a common sense understanding that any parent, older sibling, or dear friend might be able to affirm.

Previous research uncovering similar data has warned that the pleasure derived from such in-the-moment interactions might be a demonstration of enmeshment or a lack of capacity to mentalize (54). This might indeed be the case, but have we been overlooking something elemental in the creation and maintenance of wellbeing in humans who are facing arduous experiences? Social bonds characterized by mentalization, and mutual trust can be grounds for resilience. Might the therapeutic relationship be a mechanism of healing [for the youth] *and* a key element of worker wellbeing (16)? These findings support greater investment in understanding how mentalization-based therapy and intersubjectivity theory might interact as a tool towards increasing RCW wellbeing (55–57).

A second interesting result in this study was the profound power of RCW personal experience in creating a secure bond. Specifically, depicted by how one RCW enlisted their own childhood memories to care for the child in her care. Previous work has drawn attention to the power of memories to serve as triggers for countertransference (58). This study raises the question of memories, including rituals, histories, and experiences of care, as a potent source of relational strength, security, connectedness, and precious tenderness. That children need stability and predictability from their caregivers is well established, less attention has been paid to their need for RCW to open their hearts and lives to them. By listening to children share their experiences of placement, Côté and Clément (59) introduced the notion that children and youth in residential care must be anchored both physically to a secure and stable placement setting, but also anchored affectively to their RCW through love and affection.

This objective must be at the heart of the organizations' practices if children are to develop the capacity to love themselves and others and become adults capable of developing romantic and parental relationships that are healthy and rich. Children's attachment and anchorage are crucial factors for increasing resilience and reducing the risk of intergenerational repetition. p. 9

Our investigation of RCW experiences of pride and accomplishment uncovered unexpected results. Like the children in Côté and Clément's study, the RCW in our study also feel good when they were affectively anchored to the children and youth in their care. These findings suggest that organizational climates, child welfare and youth justice systems and structures must promote and provide the therapeutic space for a deeply felt sense of connectedness between those in care and those caring for them, for love and affection to flourish.

This study contributes to practice and research by describing a novel way of understanding the mechanisms underlying RCW felt sense of work-related pride and accomplishment. In doing so, it expands the discussion of protective factors against RCW work-related harms to include context dependant, complex, dynamic, relational experiences. This perspective may help organizations to identify and provide the conditions necessary for meaningful relationships between RCW and those in their care. It might also assist RCW to develop these types of relationships by prompting further reflections about how they come to feel pride and accomplishment in their work. Further research and theorizing are indicated to better understand how the relational, vicarious, and personal mechanisms underlying RCW pride and accomplishment might be related to self and other mentalization capacities, intersubjective experiences, secure attachment, resilience, factors promoting employee retention or contributing to employee turnover, organizational climate, services, and outcomes in residential care. Future work examining the impacts of worker wellbeing in promoting secure attachment bonds between RCW and those in their care, and how RCW personal histories of secure attachment can be leveraged to extend in their helping relationships are also indicated.

Several limitations should be considered when reading this work. This study reports on the exploration of experiences among a homogenous sample of RCW. Diverse sociodemographic factors such as race, ethnicity and culture are not represented as the sample is largely Caucasian and French-Canadian. Three interviewers conducted these interviews; thus, their personal styles and attributes may have influenced the data collected.

## Conclusion

The results of this study reveal the impacts of and reciprocal nature of relationships between RCW and the children and youth in their care. They described engagement in intersubjective exchanges—that can impact multiple domains of development and experience. RCW experienced personal pride and accomplishment relationally through the reciprocal connections that they shared; vicariously, through the growth and accomplishments of the children, youth, and families with whom they worked closely; and, personally, through their own success in promoting the wellbeing of a child or youth. The emergence of pride and accomplishment was most present through the quality of the connections between these professionals and the children and youth in their care.

## Data Availability

The datasets presented in this article are not readily available because the dataset contains potentially identifying data. Requests to access the datasets should be directed to delphine.collin-vezina@mcgill.ca.

## References

[1] Brend D. Residential childcare workers in child welfare and moral distress. Child Youth Serv Rev. (2020) 119:105621. 10.1016/j.childyouth.2020.105621

[2] Kim H Kao D. A meta-analysis of turnover intention predictors among U.S. Child welfare workers. Child Youth Serv Rev. (2014) 47:214–23. 10.1016/j.childyouth.2014.09.015

[3] McElvaney R Tatlow-Golden M. A traumatised and traumatising system: professionals’ experiences in meeting the mental health needs of young people in the care and youth justice systems in Ireland. Child Youth Serv Rev. (2016) 65:62–9. 10.1016/j.childyouth.2016.03.017

[4] Seti C. Causes and treatment of burnout in residential child care workers: a review of the research. Resid Treat Child Youth. (2008) 24(3):197–229. 10.1080/08865710802111972

[5] World Health Organization [WHO]. International Classification of Diseases, Eleventh Revision (ICD-11). Geneva, Switzerland: World Health Organization (WHO) (2019/2021). Available online at: https://icd.who.int/en

[6] Bride B Jones J MacMaster S. Correlates of secondary traumatic stress in child protective services workers. J Evid Based Soc Work. (2007) 4(3–4):69–80. Available online at: http://www.scopus.com/inward/record.url?eid=2-s2.0-34548639213&partnerID=40&md5=7e05b1bbac95c9cb93a5be196d237506 10.1300/J394v04n03_05

[7] Pearlman L Saakvitne K. Trauma and the Therapist: Countertransference and Vicarious Traumatization in Psychotherapy with Incest Survivors. New York, USA: WW Norton & Company (1995).

[8] Boyas J Wind L. Employment-based social capital, job stress, and employee burnout: a public child welfare employee structural model. Child Youth Serv Rev. (2010) 32(3):380–8. 10.1016/j.childyouth.2009.10.009

[9] He A Phillips J Lizano E Rienks S Leake R. Examining internal and external job resources in child welfare: protecting against caseworker burnout. Child Abuse Negl. (2018) 81:48–59. 10.1016/j.chiabu.2018.04.013 29715606

[10] Lizano E Mor Barak M. Job burnout and affective wellbeing: a longitudinal study of burnout and job satisfaction among public child welfare workers. Child Youth Serv Rev. (2015) 55:18–28. 10.1016/j.childyouth.2015.05.005

[11] Baugerud G Vangbaek S Melinder A. Secondary traumatic stress, burnout and compassion satisfaction among Norwegian child protection workers: protective and risk factors. Br J Soc Work. (2018) 48(1):215–35. 10.1093/bjsw/bcx002

[12] Craun S Bourke M. Is laughing at the expense of victims and offenders a red flag? Humor and secondary traumatic stress. J Child Sex Abus. (2015) 24(5):592–602. 10.1080/10538712.2015.1042187 26301442

[13] Edmonds K. Residential Counselor Turnover: The Effects of Burnout, Vicarious Trauma, Environment, and Self-Care. Minneapolis, USA: Doctoral dissertation, Walden University (2019).

[14] Molnar B Fraser J. Child abuse workforce health: research to promote a healthy and resilient child abuse & neglect workforce. Child Abuse Negl. (2020) 110:104704. 10.1016/j.chiabu.2020.104704 32948321

[15] Rochelle S Buonanno L. Charting the attitudes of county child protection staff in a post-crisis environment. Child Youth Serv Rev. (2018) 86:166–75. 10.1016/j.childyouth.2018.01.032

[16] Russ E Lonne B Lynch D. Increasing child protection workforce retention through promoting a relational-reflective framework for resilience. Child Abuse Negl. (2020) 110:104245. 10.1016/j.chiabu.2019.104245 31784023

[17] Sage C Brooks S Greenberg N. Factors associated with type II trauma in occupational groups working with traumatised children: a systematic review. J Ment Health. (2018) 27(5):457–67. 10.1080/09638237.2017.1370630 28898109

[18] Salloum A Choi M Stover C. Exploratory study on the role of trauma-informed self-care on child welfare workers’ mental health. Child Youth Serv Rev. (2019) 101:299–306. 10.1016/j.childyouth.2019.04.013

[19] Yoon S Yang J Pei F Benavides J Bayar Ö Logan J Can resilience change over time? Patterns and transitions in resilience among young children involved with the child welfare system. Child Dev. (2024) 95(1):191–207. 10.1111/cdev.13980 37551445 PMC10841190

[20] Bonanno G. The resilience paradox. Eur J Psychotraumatol. (2021) 12(1):1942642. 10.1080/20008198.2021.1942642 34262670 PMC8253174

[21] Kalergis K Anderson D. Lessons from the field: an evidence-informed resiliency model for child abuse organizations. Child Abuse Negl. (2020) 110:104266. 10.1016/j.chiabu.2019.104266 31735368

[22] Ungar M. Multisystemic Resilience: Adaptation and Transformation in Contexts of Change. Oxford, UK: Oxford University Press (2021). 10.1093/oso/9780190095888.001.0001

[23] Molnar B Meeker S Manners K Tieszen L Kalergis K Fine J Vicarious traumatization among child welfare and child protection professionals: a systematic review. Child Abuse Negl. (2020) 110:104679. 10.1016/j.chiabu.2020.104679 32826062

[24] Barford S Whelton W. Understanding burnout in child and youth care workers. Child Youth Care Forum. (2010) 39(4):271–87. 10.1007/s10566-010-9104-8

[25] Ribeiro da Silva D Rijo D Salekin R. Psychopathic traits in children and youth: the state-of-the-art after 30 years of research. Aggress Violent Behav. (2020) 55:1–16. 10.1016/j.avb.2020.101454

[26] Brewin C Andrews B Valentine J. Meta-analysis of risk factors for posttraumatic stress disorder in trauma-exposed adults. J Consult Clin Psychol. (2000) 68(5):748–66. 10.1037/0022-006X.68.5.748 11068961

[27] Smith C Freyd J. Dangerous safe havens: institutional betrayal exacerbates sexual trauma. J Trauma Stress. (2013) 26(1):119–24. 10.1002/jts.21778 23417879

[28] Li Y Huang H Chen Y. Organizational climate, job satisfaction, and turnover in voluntary child welfare workers. Child Youth Serv Rev. (2020) 119:105640. 10.1016/j.childyouth.2020.105640

[29] Rycraft JR. The party isn't over: the agency role in the retention of public child welfare caseworkers. Soc Work. (1994) 39(1):75–80. PMID: . 8310324

[30] Mor Barak M Levin A Nissly J Lane C. Why do they leave? Modeling child welfare workers’ turnover intentions. Child Youth Serv Rev. (2006) 28(5):548–77. 10.1016/j.childyouth.2005.06.003

[31] Glisson C Green P Williams N. Assessing the organizational social context (OSC) of child welfare systems: implications for research and practice. Child Abuse Negl. (2012) 36(9):621–32. 10.1016/j.chiabu.2012.06.002 22980071

[32] Glisson C Hemmelgarn A. The effects of organizational climate and interorganizational coordination on the quality and outcomes of children’s service systems. Child Abuse Negl. (1998) 22(5):401–21. 10.1016/S0145-2134(98)00005-2 9631252

[33] Glisson C Williams N. Assessing and changing organizational social contexts for effective mental health services. Annu Rev Public Health. (2015) 36:507–23. 10.1146/annurev-publhealth-031914-122435 25785894

[34] Glisson C Dukes D Green P. The effects of the ARC organizational intervention on caseworker turnover, climate, and culture in children’s service systems. Child Abuse Negl. (2006) 30(8):855–80. 10.1016/j.chiabu.2005.12.010 16930699

[35] Glisson C Green P. The effects of organizational culture and climate on the access to mental health care in child welfare and juvenile justice systems. Adm Policy Ment Health. (2006) 33(4):433–48. 10.1007/s10488-005-0016-0 16240076

[36] Glisson C Green P. Organizational climate, services, and outcomes in child welfare systems. Child Abuse Negl. (2011) 35(8):582–91. 10.1016/j.chiabu.2011.04.009 21855998

[37] Brown S. A meta-analysis and review of organizational research on job involvement. Psychol Bull. (1996) 120(2):235. 10.1037/0033-2909.120.2.235

[38] Glisson C. The organizational context of children’s mental health services. Clin Child Fam Psychol Rev. (2002) 5:233–53. 10.1023/A:1020972906177 12495268

[39] Glisson C Durick M. Predictors of job satisfaction and organizational commitment in human service organizations. Adm Sci Q. (1988) 33:61–81. 10.2307/2392855

[40] Glisson C James L. The cross-level effects of culture and climate in human service teams. J Organ Behav. (2002) 23(6):767–94. 10.1002/job.162

[41] de Guzman A Carver-Roberts T Leake R Rienks S. Retention of child welfare workers: staying strategies and supports. J Public Child Welf. (2020) 14(1):60–79. 10.1080/15548732.2019.1683121

[42] Strolin-Goltzman J Breslend N Hemenway Deaver A Wood V Woodside-Jiron H Krompf A. Moving beyond self-care: exploring the protective influence of interprofessional collaboration, leadership, and competency on secondary traumatic stress. Traumatology (Tallahass Fla). (2020) 30:69–76. 10.1037/trm0000244

[43] L’heureux F Truchot D Borteyrou X Rascle N. The maslach burnout inventory–human services survey (MBI-HSS): factor structure, wording effect and psychometric qualities of known problematic items. Trav Hum. (2017) 80(2):161–86. 10.3917/th.802.0161

[44] Branta H Jacobson T Alvinius A. Professional pride and dignity? A classic grounded theory study among social workers. Qual Life Qual Work Life. (2017) 285:285–98. 10.5772/68018

[45] Itzick M Kagan M Ben-Ezra M. Social worker characteristics associated with perceived meaning in life. J Soc Work. (2018) 18(3):326–47. 10.1177/1468017316654345

[46] Montross-Thomas L Scheiber C Meier E Irwin S. Personally meaningful rituals: a way to increase compassion and decrease burnout among hospice staff and volunteers. J Palliat Med. (2016) 19(10):1043–50. 10.1089/jpm.2015.0294 27337055 PMC6453491

[47] Luyten P Fonagy P. Mentalizing and trauma. Handbook of Mentalizing in Mental Health Practice. Washington, DC: American Psychiatric Association Publishing (2019) 2:79–99.

[48] Schofield G Beek M (2018). Providing a secure base. Norwich (Norfolk), UK: University of East Anglia. Available online at: https://www.uea.ac.uk/documents/96135/4784540/E9+-+Final+Secure+base+interview+17Feb21.pdf/74cf67e9-a0e4-eb00-331b-ea0f2d0792a9?t=1619011858977

[49] Thompson Burdine J Thorne S Sandhu G. Interpretive description: a flexible qualitative methodology for medical education research. Med Educ. (2021) 55(3):336–43. 10.1111/medu.14380 32967042

[50] Thorne S. Interpretive Description: Qualitative Research for Applied Practice (Second ed.). London, UK: Routledge (2016).

[51] QSR International. Release notes (NVivo 14 Mac). (2023). Available online at: https://techcenter.qsrinternational.com/Content/nm14/nm14_release_notes.htm (Accessed June 1, 2024).

[52] Braun V Clarke V. To saturate or not to saturate? Questioning data saturation as a useful concept for thematic analysis and sample-size rationales. Qual Res Sport Exerc Health. (2021) 13(2):201–16. 10.1080/2159676X.2019.1704846

[53] Spinazzola J van der Kolk B Ford J. Developmental trauma disorder: a legacy of attachment trauma in victimized children. J Trauma Stress. (2021) 34(4):711–20. 10.1002/jts.22697 34048078 PMC8453773

[54] Modlin H Magnuson D. A constructive-developmental analysis of satisfaction, challenge and coping in residential child and youth care. Child Youth Serv. (2021) 42(2):179–99. 10.1080/0145935X.2021.1903857

[55] Bateman A Fonagy P. Mentalization-Based treatment. Psychoanal Inq. (2013) 33(6):595–613. 10.1080/07351690.2013.835170 26157198 PMC4467231

[56] Jacobsen M Ha C Sharp C. A mentalization-based treatment approach to caring for youth in foster care. J Infant Child Adolesc Psychother. (2015) 14(4):440–54. 10.1080/15289168.2015.1093921

[57] Rasmussen B. An intersubjective perspective on vicarious trauma and its impact on the clinical process. J Soc Work Pract. (2005) 19(1):19–30. 10.1080/02650530500071829

[58] St. Jean S Rasmussen B Gillespie J Salhani D. Today in light of yesterday: an exploration of Workers’ childhood memories in the context of child protection practice. Br J Soc Work. (2021) 51:1150–67. 10.1093/bjsw/bcab011

[59] Côté C Clément M. Let’s talk about love: perceptions of children in residential care. Child Youth Serv Rev. (2022) 140:1–11. 10.1016/j.childyouth.2022.106584

